# Safety and Efficacy of Intravenous Ferric Carboxymaltose (750 mg) in the Treatment of Iron Deficiency Anemia: Two Randomized, Controlled Trials

**DOI:** 10.1155/2012/172104

**Published:** 2012-09-10

**Authors:** Charles F. Barish, Todd Koch, Angelia Butcher, David Morris, David B. Bregman

**Affiliations:** ^1^Wake Gastroenterology and Wake Research Associates, Raleigh, NC 27612, USA; ^2^Luitpold Pharmaceuticals, Inc., Norristown, PA 19403, USA; ^3^WebbWrites, LLC, Durham, NC 27705, USA; ^4^Department of Pathology, Albert Einstein College of Medicine, NY 10461, USA

## Abstract

*Background*. Iron deficiency anemia (IDA) is a common hematological complication with potentially serious clinical consequences that may require intravenous iron therapy. Ferric carboxymaltose (FCM) is a stable, nondextran iron formulation administered intravenously in large single doses to treat IDA. *Objective*. Two open-label, randomized, placebo-controlled trials evaluated safety of multiple or single 750 mg FCM doses compared to standard medical care (SMC) in IDA patients. Secondary endpoints were improvements in hemoglobin and iron indices. *Design and Patients*. Adults with hemoglobin ≤12 g/dL, ferritin ≤100 or ≤300 ng/mL with transferrin saturation ≤30% were randomized to receive single (*n* = 366) or weekly (*n* = 343) FCM or SMC (*n* = 360 and *n* = 366). *Results*. Significantly greater (*P* ≤ 0.001) increases in hemoglobin and iron indices occurred in FCM groups versus SMC. In the multidose study, up to two infusions of FCM were needed to reach target iron levels versus 3–5 of intravenous iron comparators. FCM and SMC groups had similar incidences and types of adverse events and serious adverse events. Transient hypophosphatemia not associated with adverse events or clinical sequelae occurred in the FCM groups. *Conclusion*. Intravenous FCM is safe, well tolerated, and associated with improvements in hemoglobin and iron indices comparable to SMC when administered in single doses of up to 750 mg at a rate of 100 mg/min. Fewer FCM infusions were required to reach target iron levels compared to other intravenous iron preparations.

## 1. Introduction

 Iron deficiency anemia (IDA) is a common hematological complication with a prevalence of 2% among adult men and 9–20% among adult women depending on race and ethnicity [1]. IDA occurs in several medical conditions including inflammatory bowel disease (IBD) and chronic kidney disease (CKD), in association with cancer and its treatment, and in cases of acute blood loss resulting from gastrointestinal (GI) bleeding, trauma, surgery, and obstetrics/gynecology conditions [2]. IDA contributes significantly to morbidity, disease burden, and decreased quality of life [3–5].

 While oral iron is typically considered first-line treatment, high-dose intravenous (IV) iron therapy has a role in the treatment of varied clinical situations associated with IDA. IV iron is more effective in patients with IBD [6] and in oncology patients receiving erythropoietin-stimulating agents (ESAs) for chemotherapy-induced anemia [7]. IV iron is also preferred when iron requirements are too high to be corrected with oral iron therapy (e.g., gastrointestinal bleeding), when oral iron cannot be properly absorbed (e.g., after gastric bypass or in celiac disease), and when oral iron is not well tolerated because of gastrointestinal side effects. Approximately 10–20% of patients taking oral iron experience nausea, constipation, epigastric discomfort, and vomiting that may lead to noncompliance [8]. IV iron is recommended for hemodialysis-dependent CKD patients due to frequent blood losses on dialysis machines and difficulty utilizing oral iron [9, 10] and is also an option for treatment of predialysis CKD patients [9].

 IV iron formulations available in the United States (US) vary in dosing regimens and indications and may be limited by the maximum dose that can be given in a single visit due to instability of the iron-carbohydrate moiety (e.g., iron sucrose and iron gluconate formulations) as well as by anaphylactic reactions due to dextran-containing iron formulations [11, 12]. Feraheme (ferumoxytol) is a recently approved IV iron product that can be administered as two injections of 510 mg [12]. Feraheme contains a modified dextran shell designed to reduce immunogenic potential, however anaphylaxis in the setting of previous hypersensitivity to iron dextran has been reported [13].

 Ferric carboxymaltose (FCM) is a stable, non-dextran-containing iron formulation that permits the uptake of iron by the reticuloendothelial system with minimal release of free iron [14]. FCM was developed for rapid IV administration in high doses for the treatment of iron deficiency [15, 16] and the rapid infusion of up to 1000 mg of FCM over 15 min has been shown to be well tolerated. The FCM complex has a nearly neutral pH (5.0–7.0) with a physiologic osmolarity and no dextran cross-reactivity [17]. The iron-carbohydrate FCM complex is more stable than ferric gluconate or iron sucrose, permitting slow and controlled delivery of iron into target tissues.

 Numerous clinical trials in over 2000 patients have shown that FCM improves hemoglobin levels and replenishes depleted iron stores [18]. FCM is effective and well tolerated in patients with IDA associated with a variety of medical conditions including IBD, CKD, heavy uterine bleeding, and postpartum anemia [19–23]. FCM treatment also improves symptoms, functional capacity, and quality of life in patients with chronic heart failure and iron deficiency with or without anemia [24]. FCM is approved in Europe for the treatment of iron deficiency when oral iron preparations are ineffective or cannot be used.

 The primary objective of the present studies was to evaluate the safety of FCM at doses up to 750 mg compared to standard medical care (SMC) in patients with IDA.

## 2. Methods

Two phase 3, open-label, randomized, controlled, multicenter trials (NCT00703937 and NCT00704353) were conducted from July 2008 to July 2009 in 95 centers each. Study protocols (1VIT08019 and 21, Luitpold Pharmaceuticals) and informed consent forms were approved by institutional review boards of each center, and trials complied with the declaration of Helsinki. Enrolled subjects provided informed consent. Study 1VIT08019 was a FCM multidose study and Study 1VIT08021 was an FCM single-dose study. In both studies patients were randomized to either FCM or standard medical care (SMC). For patients in the SMC group, the treating physician could select approved oral or IV iron therapy thus permitting head-to-head comparisons to IV iron as well as oral iron.

### 2.1. Setting and Participants

Men and women 18 to 85 years of age with anemia attributable to iron deficiency were eligible if they were not dialysis dependent and had screening laboratory values for hemoglobin ≤11 (multidose study) or ≤12 g/dL (single-dose study), and ferritin ≤100 ng/mL or ≤300 ng/mL if transferrin saturation (TSAT) was ≤30%. Patients received study treatments on an outpatient basis.

Exclusion criteria included known hypersensitivity to any component of FCM, hemochromatosis or iron storage disorders, current treatment for asthma, recent treatment with IV iron, antibiotics or red blood cell transfusion, evidence of infection (including hepatitis B, hepatitis C, or HIV antibodies), concomitant severe cardiovascular diseases, pregnant or lactating women and women of child-bearing potential not using approved birth control methods, or any condition which, in the opinion of the investigator, made participation unacceptable.

### 2.2. Randomization and Interventions

Subjects underwent medical history and clinical evaluation during the screening phase and were randomized within 7 days to the treatment phase (Day 0) by a centralized interactive voice-response system if entry criteria were met. Randomization was stratified by baseline hemoglobin (≤8, 8.1 to 9.5, ≥9.6 g/dL), cardiovascular-disease risk (category 1 to 4 based on variables included in the Framingham risk model [25] including smoking status, prior history of cardiovascular disease, use of low-dose aspirin, and whether patients had diabetes, hypertension, or hyperlipidemia, where 1 is no known risk factor and 4 is a prior history of cardiovascular disease), use of immunosuppressive therapy, and past response to oral iron (poor/yes or poor/no). Subjects were randomized in a 1 : 1 ratio to receive FCM or SMC (including oral iron, IV iron, or no iron replacement) for treatment of IDA.

For the single-dose study, subjects received 750 mg FCM or 15 mg/kg, whichever was smaller, administered by IV push injection at 100 mg per minute on Day 0. For the multidose study, subjects received undiluted FCM 15 mg/kg up to a single dose of 750 mg at 100 mg per minute weekly until the calculated iron deficit dose had been administered (to a maximum cumulative dose of 2,250 mg). The FCM dose was determined by calculating the total iron requirement and dividing this total amount into one or more single dose(s) according to the Ganzoni formula: Total iron deficit (mg) = Body weight (kg) × (Target Hb − Actual Hb) (g/dL) × 2.4 + depot iron of 500 mg.

Subjects randomized to the SMC group received SMC from Day 0–30 for the single-dose study and Day 0−42 for the multidose study. Type of SMC was determined and documented by the investigator, and included approved oral or IV iron preparations. Feraheme [ferumoxytol] [12] is an IV iron formulation that was approved in June, 2009 (at about the time that these trials finished enrolling), and was therefore not included in the SMC options.

### 2.3. Outcomes and Followup

Clinical, laboratory, and safety data were collected at study visits on Days 0, 7 and 30/end-of-treatment visit for the single-dose study and Days 0, 7, 14, 28, and 42/end-of-study visit for the multidose study. SMC subjects and subjects terminating early had a follow-up phone call 28–30 days after study-drug administration.

### 2.4. Statistical Analyses

The primary objective of both studies was to evaluate the safety of a maximum administered dose of 750 mg of FCM compared to SMC in the treatment of IDA. The safety population included all subjects administered study medication. Adverse events were reported from Day 0 to the completion of the study.

The multidose study included efficacy analyses that were based on a modified intent-to-treat (mITT) population consisting of all subjects from the safety population who had 2 baseline hemoglobin values (with less than a 1-gram difference between the 2) and at least 1 postbaseline hemoglobin assessment. Efficacy analyses were summarized for the following treatment groups:

- FCM.
- Venofer (iron sucrose injection, USP) and Ferrlecit (sodium ferric gluconate).
- Oral iron.
- Other treatment.

Clinically meaningful hemoglobin increases were defined for the different patient populations: CKD ≥ 1 g/dL; heavy uterine bleeding or GI ≥ 2 g/dL; postpartum ≥ 3 g/dL; other ≥ 2 g/dL. The proportion of subjects in the mITT Population achieving a hemoglobin increase that was clinically meaningful for that population anytime between baseline and end of study or time of intervention was summarized for each therapy group. A 95% 2-sided confidence interval for the difference between FCM and each of the other therapy groups was based on the normal approximation for the binomial distribution, using the Wald continuity correction [(1/*n*_1_ + 1/*n*_2_)/2]. Logistic regression was used to provide a confidence interval for the comparison of FCM and each of the other therapy groups after adjusting for important baseline differences and potential baseline predictors of efficacy response.

 No efficacy analyses were conducted in the single-dose study, although hemoglobin levels and other hematology indices were collected as part of the clinical chemistry and laboratory assessments.

 A sample size of 500 subjects per group in each trial was estimated to provide approximately 80% power to detect a statistically significant difference with Fisher's exact test in the incidence of serious adverse events. However, by pooling the results of the two trials approximately 90% power could be achieved with a pooled sample size of approximately 700. Hence enrollment was stopped when 708 and 736 subjects were enrolled in the multidose and single-dose studies, respectively.

 For baseline characteristics, treatment group differences for categorical characteristics (e.g., sex, race) were assessed with Fisher's exact test and quantitative characteristics (e.g., age, weight) were assessed using the *t*-test for independent groups. For safety assessments, comparison of incidence rates was performed with the 2-sided Fisher's exact test. Comparisons of continuous endpoints, such as mean change in clinical laboratory findings were performed using the one-way analysis of variance (ANOVA) with a factor for randomized treatment group. The number of subjects with missing data was tabulated but excluded from Fisher's exact test. No formal statistical tests were performed for the incidence of abnormal laboratory values or vital signs.

## 3. Results

**Figure 1:**
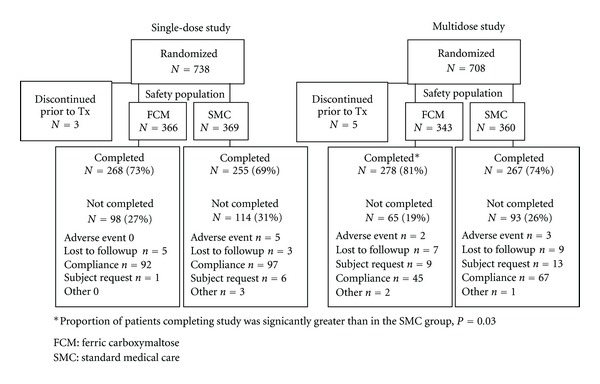
Disposition of subjects in the single-dose and multidose studies.

Seven hundred and thirty-eight patients were randomized in the single-dose study (366 in the FCM group, and 369 in the SMC group, 3 not treated) and 708 (343 in the FCM group and 360 in the SMC group, 5 not treated) in the multidose study. Disposition and reasons for not completing the studies are shown in Figure 1. The majority of subjects completed the studies. In the multidose study, the proportion of subjects completing the study in the FCM group was significantly greater than in the SMC group (*P* = 0.03).

No clinically significant differences were observed between the FCM and SMC groups for any demographic or baseline characteristic in either study (summarized in Table 1). Both studies were comprised primarily of women. Mean hemoglobin, TSAT, and ferritin values at screening were similar between the FCM and SMC groups. The majority of subjects were not currently using ESA, had received previous iron therapy, had no history of iron intolerance or drug allergy, had not used immunosuppressive therapy, and had a poor/no response to previous iron therapy. The cardiovascular disease risk for patients was primarily category 1 or 2, indicating the patient had no risk factors (category 1) or a single risk factor (category 2). The primary etiology of IDA included CKD, heavy uterine bleeding, gastrointestinal conditions, and postpartum causes. Just over half the subjects received oral iron. Among subjects receiving IV iron as SMC, Venofer and iron dextran were most commonly used.

**Figure 2:**
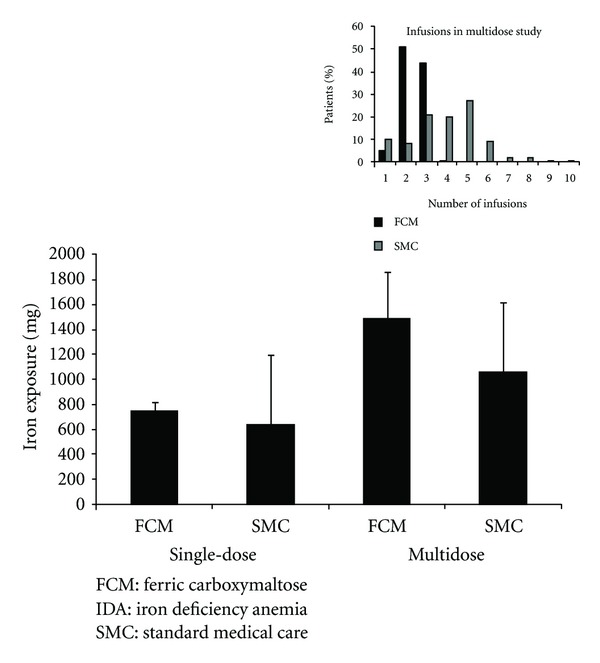
Extent of exposure to IV iron. Mean exposure with standard deviations for the FCM and SMC groups in the single-dose and multidose studies. The mean maximum single infusion in the multidose study was 745.7 ± 45.1 mg in the FCM group and 380.1 ± 407.2 mg in the SMC group. The insert shows the number of infusions administered in the multidose study.

The total doses of IV iron (mg) in either the FCM or the SMC groups are shown in Figure 2. In the single-dose study, the extent of iron exposure was similar in the FCM and SMC groups, although there was more variation in the SMC group. In the multidose study, the mean maximum single infusion in the FCM group was close to twice that of the SMC group (745.7 ± 45.1 mg versus 380.1 ± 407.2 mg). The number of infusions in the multidose study is also shown in Figure 2 (inset). In the FCM group, 1 or 2 infusions were needed over the course of the study to reach their target iron level, while in the SMC group, 3–5 infusions were needed.

### 3.1. Safety

Table 2 summarizes adverse event reporting in FCM and SMC groups. The overall incidences of treatment-emergent adverse events were similar across groups in both studies. In the single-dose study, the most common treatment-emergent events were nausea, vomiting constipation, diarrhea, urinary tract infection, and abdominal pain in the SMC group and nausea, headache, diarrhea, and fatigue in the FCM group. A statistically significantly greater proportion of subjects in the SMC group compared with the FCM group experienced vomiting (4% versus 1%, *P* = 0.029) and urinary tract infection (2% versus 0.3%, *P* = 0.038).

In the multidose study, common (≥5%) treatment-emergent adverse events in the SMC group were nausea, constipation, dizziness, and headache, and in the FCM group, headache. A statistically significantly greater proportion of subjects in the SMC group (7%) experienced constipation compared with those in the FCM group (4%), *P* = 0.031. A statistically significantly greater proportion in the FCM group compared to the SMC group experienced blood phosphorus decrease/hypophosphatemia (7% versus 0%, resp., *P* < 0.001) recorded by investigators as a result of clinical chemistry values below 2 mg/dL. None of the cases were considered serious or required action, and none resulted in discontinuation of a patient from the study.

The majority of treatment-emergent adverse events experienced during both studies were classified by the Investigator as Grade 1 or 2 in severity. In the single-dose study, 14 (4%) and 12 (3%) subjects in the FCM and SMC groups, respectively, experienced Grade 3 events, and 1 subject each in the SMC group experienced a Grade 4 and Grade 5 event. In the multidose study, 35 (10%) and 12 (3%) patients in the FCM and SMC groups, respectively, had Grade 3 treatment-emergent adverse events. Three subjects (0.9%) in the FCM group and 2 (0.6%) in the SMC group experienced events classified as Grade 4 severity.

In the single-dose study, there were no discontinuations in the FCM group and 6 (2%) in the SMC group due to an adverse events. In the multidose study, 17 (5%) subjects in the FCM group and 11 (3%) in the SMC group had at least 1 serious adverse events. One case in the FCM group (constipation) and 2 cases in the SMC group (renal infarct and hypotension) were considered related to study medication. In the single dose study, 5 (1%) subjects in the FCM group and 9 (2%) in the SMC group experienced at least 1 serious adverse event, none of which were considered related to study medication. Two (2; 0.6%) and 3 (0.8%) subjects in the FCM and SMC groups, respectively, were discontinued from the multidose study due to an adverse event.

There were no deaths during the multidose study. In the single-dose study, one subject in the SMC group died from pneumonia.

The only significant differences in laboratory or clinical chemistry were the proportion of subjects in the FCM groups in both studies with phosphorus levels that dropped to less than 2 mg/dL from normal baseline in the range of 2.5–4.5 mg/dL. In the single dose study, 18% of subjects in the FCM group had decreases compared to 0.3% in the SMC group. In the multidose study, 47.9% of subjects in the FCM group showed a decline compared with 0.3% in the SMC group. These decreases were not associated with any clinical sequelae.

Evaluations of vital signs and physical examinations showed no clinically meaningful differences from baseline in either the FCM or SMC groups. In the single-dose study, 3 subjects in the FCM group and 1 in the SMC group experienced a nonserious hypersensitivity/allergic reaction, and in the multidose study, 1 (0.3%) and 3 (0.8%) subjects in the FCM and SMC groups, respectively, experienced hypersensitivity during the study. The events resolved after discontinuation of study drug (one FCM subject) or administration of antihistamines and/or glucocorticoids (3 subjects, 2 FCM, and 1 SMC). In the multidose study, 5 subjects in the FCM group and 1 in the SMC group who received Venofer had reported adverse events of hypotension (Grade 1 or 2) during the study. In the single-dose study, 3 subjects in the FCM group and none in the SMC group had low-blood-pressure readings recorded on the day of dosing in the absence of symptomatic hypotension.

### 3.2. Efficacy

 In the single-dose study, statistically significantly greater (*P* ≤ 0.001) mean increases from baseline to day 30 were observed in the FCM group compared with the SMC group for hemoglobin (1.15 ± 1.13 versus 0.78 ± 1.05 g/dL) and ferritin levels (146.4 ± 111.4 versus 67.1 ± 140.2 ng/mL), and hematocrit (3.4 ± 3.6 versus 2.4 ± 3.5%). Significantly greater mean increases from baseline to day 30 occurred in the FCM group compared with the SMC group in absolute reticulocyte count (*P* = 0.016), basophils, mean corpuscular hemoglobin, mean corpuscular volume, red-cell distribution width (all *P* < 0.001), and red-blood-cell counts (*P* = 0.019). A statistically significantly greater mean decrease from baseline to Day 30 was observed in the FCM group compared to the SMC group in platelets (i.e., decreases of 55 versus 35 thousand platelets per microliter from baselines of 312 and 315 thousand platelets per microliter, resp., *P* < 0.001). There were no significant differences between the two groups in the percent eosinophils, lymphocytes, mean corpuscular hemoglobin concentration, monocytes, neutrophils, or white blood counts.

**Figure 3:**
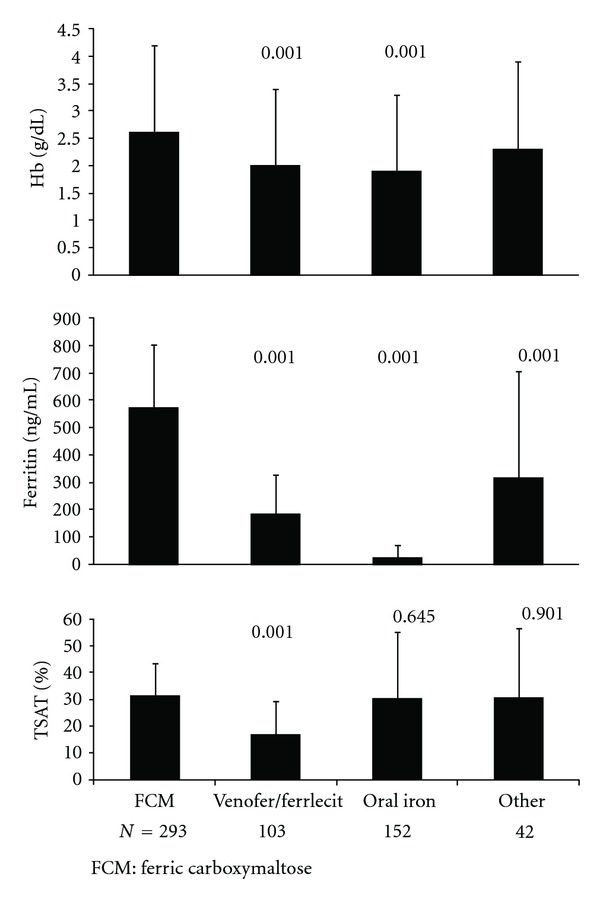
Mean changes in hemoglobin, ferritin, and TSAT from baseline to the highest value between baseline and end of study or time of intervention for subjects in the multidose study (mITT population). *P* values for the differences between SMC groups versus FCM (determined by one-way ANOVA) are shown above the bars.

 Mean changes from baseline in hemoglobin, ferritin, and TSAT are shown in Figure 3 for the mITT population in the multidose study. The mean increases from baseline to the highest value between baseline and the end of study or time of intervention in the FCM group were statistically significantly greater for hemoglobin, ferritin, and TSAT compared to subjects in the SMC group receiving Venofer or Ferrlecit, for hemoglobin and ferritin compared to the oral iron group, and for ferritin compared to other SMC treatment.

Table 3 shows the proportion of subjects in the multidose study mITT population achieving changes in hemoglobin defined in the protocol as clinically meaningful for each population of patients (chronic kidney disease [CKD] ≥ 1 g/dL; heavy uterine bleeding or gastrointestinal (GI) ≥ 2 g/dL; postpartum ≥ 3 g/dL; other ≥ 2 g/dL) anytime between baseline (day 0) and end of treatment. These results are shown for the overall study population and stratified by IDA etiology for the FCM group and SMC subgroups. There were no statistically significant differences between the FCM group and SMC subgroups. 

## 4. Discussion

 The primary objective of these studies was to assess safety of FCM compared to SMC. FCM was well tolerated, and there were no clinically important differences in safety outcomes between the FCM and SMC group. The incidences of adverse events were similar overall, and the majority of adverse events were of grade 1 or 2 severity and were consistent with medical issues in these patient populations. Importantly, there were no deaths or anaphylactic reactions in the FCM groups in either study. FCM has been registered by the public health authorities in Europe since 2007 and has been used to treat iron deficiency in over 35 countries. FCM is in clinical development in the US.

 The transient, asymptomatic hypophosphatemia observed in the FCM group in the present studies and as previously reported [20, 22, 23] is apparently not associated with adverse events or clinical outcomes in patients with IDA. Selective decreased blood phosphate levels have been reported in acute hemolytic anemia, following hematopoietic reconstitution after allogeneic peripheral blood stem cell transplantation, and after iron administration [26–28]. Decreased levels may be a reflection of cellular uptake of extracellular phosphate associated with rapid expansion of erythropoiesis [22]. It may be associated with an increase in fibroblast growth factor 23, which plays a role in phosphate homeostasis [28]. A recent case study [29] described a renal transplant patient on long-term tacrolimus therapy who developed severe and symptomatic hypophosphatemia after IV FCM administration. While it remains unclear whether the tacrolimus therapy and resulting renal tubule toxicity contributed to the complication in this transplant recipient, in general, hypophosphatemia does not appear to alter the risk/benefit profile for use of FCM, and FCM is well tolerated in patients with IDA due to chronic kidney disease [30] or other causes.

 The efficacy of FCM in treating IDA has been well documented in randomized clinical trials [19–23]. In the present studies, the mean increases from baseline to the highest value between baseline and the end of study or time of intervention for subjects in the FCM group were statistically significantly greater for hemoglobin, ferritin, and TSAT compared to subjects in the SMC group receiving Venofer or Ferrlecit, for hemoglobin and ferritin compared to the oral iron group, and for ferritin compared to other SMC treatment. Efficacy results were comparable across IDA associated with varied clinical conditions including gastrointestinal disorders, CKD, and obstetrics/gynecology conditions. The present studies did not show a statistically significant differences between FCM and SMC with respect to protocol-defined clinically meaningful increases in hemoglobin for each population (i.e., chronic kidney disease [CKD] ≥ 1 g/dL; heavy uterine bleeding or gastrointestinal (GI) ≥ 2 g/dL; postpartum ≥ 3 g/dL; other ≥ 2 g/dL) anytime between baseline (day 0) and end of treatment.

 A single dose of 750 mg of FCM can be administered at a rate of 100 mg/min, thereby permitting use of FCM on an outpatient basis, and reducing clinic visits and venipunctures resulting in fewer disruptions to patients' lifestyles. Clinically, target hemoglobin levels may be achieved more rapidly, achieving stability earlier than when using multiple small doses. In the multidose study, the majority of patients (96%) in the FCM group required 1 or 2 doses to reach their target iron levels versus 3–5 doses required for the SMC treatments. Ferritin levels were also increased with 1 or 2 doses of FCM, and the increases were significantly greater than those occurring in the SMC groups. The reduced frequency of infusions with FCM may reduce health care visits, reduce the risk of infection, and preserve vein integrity. A further potential clinical benefit of high-dose intravenous iron is to overcome the inhibition of gastrointestinal iron absorption induced by hepcidin in patients with anemia of chronic disease or other inflammatory conditions (28, 29).

 Strengths of the studies include the large sample sizes and the ability to confirm statistically and clinically significant improvements in hemoglobin indices even though several medical conditions contributing to IDA were represented in the trial. A potential weakness was the heterogeneity of the SMC comparators. Efficacy endpoints were compared to specific comparator agents (Venofer or Ferrlecit, oral iron) in some cases. Safety was compared to SMC although some adverse events would be expected to be different for oral versus intravenous comparators (e.g., constipation that is observed more prominently with oral iron).

 In conclusion, FCM at single doses of up to 750 mg was safe and effective in patients with a broad range of IDA-associated medical conditions. Overall the FCM and SMC groups were similar in terms of incidences of adverse events and serious adverse events (including deaths). The transient hypophosphatemia observed in the FCM groups was not associated with adverse events or clinical sequelae. While improvements in hemoglobin and iron indices in patients treated with FCM were comparable to other IV iron formulations, fewer infusions of FCM were needed to reach target iron levels. 

## Figures and Tables

**Table 1:** Demographics and baseline characteristics of subjects (safety population).

	Multidose study		Single-dose study	
	FCM	SMC	FCM	SMC
	(*N* = 343)	(*N* = 360)	(*N* = 366)	(*N* = 369)
Age (years)	49.3 ± 18.5	47.7 ± 17.6	49.2 ± 19.9	49.6 ± 19.9
Sex				
Female	292 (85.1)	318 (88.3)	322 (88.0)	315 (85.4)
Male	51 (14.9)	42 (11.7)	44 (12.0)	54 (14.6)
Race/Ethnicity				
Caucasian	185 (53.9)	180 (50.0)	171 (46.7)	205 (55.6)
African American	87 (25.4)	113 (31.4)	114 (31.1)	96 (26.0)
Hispanic	58 (16.9)	61 (16.9)	70 (19.1)	48 (13.0)
Asian	3 (0.9)	1 (0.3)	6 (1.6)	10 (2.7)
Other	10 (2.9)	5 (1.4)	5 (1.4)	10 (2.7)
Weight (kg)	82.2 ± 22.4	85.4 ± 22.0	83.2 ± 24.3	82.2 ± 22.9
IDA Etiology				
CKD	40 (11.7)	34 (9.4)	67 (18.3)	53 (14.4)
Uterine bleeding	77 (22.4)	103 (28.6)	61 (16.7)	79 (21.4)
GI related	69 (20.1)	66 (18.3)	49 (13.4)	47 (12.7)
Postpartum	35 (10.2)	36 (10.0)	56 (15.3)	49 (13.3)
Other/Unknown	122 (35.6)	121 (33.6)	133 (36.3)	141 (38.2)
Hb (g/dL)	9.6 ± 1.1	9.6 ± 1.1	10.8 ± 1.1	10.8 ± 1.0
Hb Category (g/dL)				
≤8.0	40 (11.7)	46 (12.8)	9 (2.5)	5 (1.4)
8.1–9.5	109 (31.8)	112 (31.1)	71 (19.4)	71 (19.2)
≥9.6	194 (56.6)	202 (56.1)	286 (78.1)	293 (79.4)
TSAT (%)	10.6 ± 7.9	10.5 ± 8.0	15.9 ± 9.2	15.0 ± 8.4
Ferritin (ng/mL)	25.6 ± 48.3	27.3 ± 66.4	38.1 ± 53.4	34.0 ± 51.1
No Current ESA use	337 (98.3)	356 (99.9)	347 (94.8)	351 (95.1)
No Iron Intolerance	283 (82.5)	302 (83.9)	333 (91.0)	318 (86.2)
Previous Iron Therapy	242 (70.6)	263 (73.1)	241 (65.8)	256 (69.4)
Response: Poor/No	208 (60.6)	217 (60.3)	246 (67.2)	248 (67.2)
No imm.supp. therapy	340 (99.1)	353 (98)	357 (97.5)	357 (96.7)
Cardiovascular risk				
≤2	201 (58.6)	222 (61.7)	217 (59.3)	230 (62.3)
>2	142 (41.4)	138 (38.3)	149 (40.7)	139 (37.7)
Type of SMC	NA		NA	
Oral Iron		187 (51.9)		210 (56.9)
IV Iron		154 (42.8)		128 (34.7)
Venofer		112 (31.1)		69 (18.7)
Iron Dextran		33 (9.2)		56 (15.2)
Ferrlecit		9 (2.5)		3 (0.8)
Other or no treatment		20 (5.6)		30 (4.6)

Values are expressed as mean ± standard deviation or number (percentage).

CKD: chronic kidney disease; ESA: erythropoiesis-stimulating agent; FCM: ferric carboxymaltose; Hb: hemoglobin; IDA: iron deficiency anemia; IV: intravenous; NA: not applicable; SMC: standard medical care; TSAT: transferring saturation.

**Table 2:** Incidence of treatment-emergent adverse events occurring in ≥2% of subjects.

	Single-dose study		
	*N* = 735		*P* value
	FCM	SMC	
	*N* = 366	*N* = 369	
≥1 event	132 (36.0)	128 (34.6)	
Nausea	15 (4.1)	18 (4.9)	0.722
Headache	11 (3.0)	5 (1.4)	0.138
Diarrhea	10 (2.7)	8 (2.2)	0.642
Fatigue	10 (2.7)	7 (1.9)	0.474
Constipation	7 (1.9)	13 (3.5)	0.257
Vomiting	4 (1.1)	14 (3.8)	0.029*
Abdominal pain	2 (<1)	8 (2.2)	0.107
Urinary tract infection	1 (<1)	8 (2.2)	0.038*

	Multidose study		
	*N* = 703		
	FCM	SMC	
	*N* = 343	*N* = 360	

≥1 event	185 (54.4)	198 (55.0)	
Decreased blood phosphorous	25 (7.3)	0	<0.001**
Headache	16 (4.7)	18 (5.0)	0.862
Nausea	14 (4.1)	27 (7.5)	0.055
Constipation	12 (3.5)	26 (7.2)	0.031*
Diarrhea	10 (2.9)	14 (3.9)	0.537
Dizziness	10 (2.9)	17 (4.7)	0.242
Upper respiratory tract infection	9 (2.6)	6 (1.7)	0.440
Arthralgia	8 (2.3)	8 (2.2)	1.000
Myalgia	8 (2.3)	2 (0.06)	0.058
Pain in extremity	9 (2.6)	4 (1.1)	0.167
Fatigue	9 (2.6)	5 (1.4)	0.287
Increased alanine aminotrasferase	7 (2.0)	5 (1/4)	0.570
Back pain	7 (2.0)	6 (1.7)	0.784
Nasopharyngitis	7 (2.0)	6 (1.7)	0.784
Urinary tract infection	7 (2.0)	6 (1.7)	0.784
Vomiting	6 (1.7)	13 (3.6)	0.164
Peripheral edema	3 (<1)	8 (2.2)	0.224
Cough	2 (<1)	8 (2.2)	0.108

Values are expressed as number of subjects (percentage).

FCM: ferric carboxymaltose; SMC: standard medical care.

*Statistically significant at 0.05 level.

**Statistically significant at 0.001 level.

**Table 3:** Proportion of subjects in the multidose study with a clinically meaningful increase in hemoglobin anytime between baseline and the end of study or time of intervention.

		SMC subgroup		
	FCM	Venofer or Ferrlecit	Oral iron	Other treatment
	*N* = 293	*N* = 103	*N* = 152	*N* = 42
	*n* (%)			
	187 (64)	59 (57)	68 (45)	22 (52)
Difference from FCM 95% CI		0.07 (−0.05, 0.18)	0.19 (0.09, 0.29)	0.11 (−0.06, 0.29)
	*n*/*N* (%)			

IDA Etiology				
CKD	15/34 (44)	8/16 (50)	2/11 (18)	1/4 (25)
Heavy uterine bleeding	55/71 (78)	16/25 (64)	25/52 (48)	8/11 (73)
IBD/GI related	41/61 (67)	19/25 (76)	4/15 (27)	7/15 (47)
Postpartum	16/24 (67)	0	8/18 (44)	1/1 (100)
Other/Unknown	60/103 (58)	16/37 (43)	29/56 (52)	5/11 (45)

FCM: ferric carboxymaltose; IDA: iron deficiency anemia; SMC: standard medical care.
